# Children’s Health: Child Survival Gets TV Boost

**Published:** 2005-10

**Authors:** Adrian Burton

A recently launched campaign known as Rx for Child Survival gets into top gear 1–3 November 2005 with the help of a six-part TV series on global health narrated by actor Brad Pitt. The series, *Rx for Survival—A Global Health Challenge*, which was produced by WGBH/NOVA Science Unit and Vulcan Productions and will be aired by PBS stations nationwide, aims to help Americans better understand global health problems. The series also highlights the plight of children under age 5, of whom more than 6 million die every year in the developing world from diseases that could be prevented or treated for just a few dollars.

The TV series is part of a multimedia project that also includes the efforts of *Time* magazine, Penguin Press, NPR, and an interactive website (http://www.pbs.org/wgbh/rxforsurvival/campaign/index.html). Says Paula Apsell, senior executive producer for WGBH/NOVA, “With the power of television to extend our message into eighty-six million living rooms each week, one of the most visited dot-org websites in the world, the local reach of three hundred forty-eight member stations across the U.S., and a far-reaching impact campaign forged on the precept of partnership, PBS is in a unique position to help Americans learn more about the world’s most pressing issues and to show them ways to do more to make the world a better place.”

With funds initially provided by the Bill & Melinda Gates Foundation and The Merck Company Foundation, the campaign’s goal is not just to inform but also to encourage Americans to donate and raise money for public health interventions in the world’s poorest countries. Some 88 cents of every dollar raised will be spent in the field to provide vaccines against measles and tetanus, insecticide-treated netting to prevent malaria, vitamin supplementation, oral rehydration packs for diarrhea (which kills 1–4 million children per year), antibiotics, and anti-malarial agents.

The actual field work will be performed by CARE and Save the Children, humanitarian organizations with delivery infrastructures already in place. The initial recipient countries will be Afghanistan, Mali, Mozambique, Nepal, Nicaragua, Sierra Leone, and Vietnam.

“Simple and affordable tools exist to save six million young lives lost each year from preventable causes like diarrhea and pneumonia. But these tools are not reaching the children who need them most,” says Charles MacCormack, CEO of Save the Children. This is because humanitarian groups cannot afford to buy and distribute them. “Through Rx for Child Survival, Americans can help these children survive and thrive,” says MacCormack.

Why another fundraising initiative? The answer is simple, says Jorge Alvar, head of the World Health Organization Department of Communicable Disease Control: “Children are dying, and we can prevent much of this tragedy at just a few dollars per head. This campaign, which aims to inform and mobilize Americans—citizens of the world’s richest nation—could be of great help in that task.”

## Figures and Tables

**Figure:**
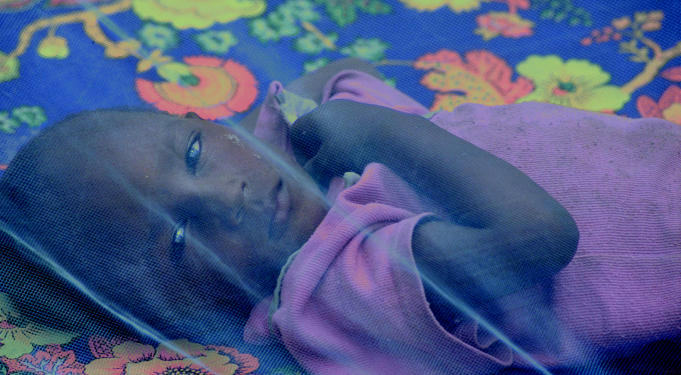
Hope for better things. The Rx for Child Survival campaign is a bid to better child health world-wide using simple tools such as insecticide-treated netting to combat malaria-bearing mosquitoes.

